# Right-sided approach to left bundle branch area pacing combined with atrioventricular node ablation in a patient with persistent left superior vena cava and left bundle branch block: a case report

**DOI:** 10.1186/s12872-022-02914-0

**Published:** 2022-11-05

**Authors:** Tine Prolič Kalinšek, David Žižek

**Affiliations:** 1 Department of Cardiology, University Medical Centre Ljubljana, Zaloška cesta 7, 1000 Ljubljana, Slovenia; 2 Faculty of Medicine, University of Ljubljana, Ljubljana, Slovenia

**Keywords:** Left bundle branch area pacing, Conduction system pacing, Pace and ablate, Left bundle branch block

## Abstract

**Background:**

Left bundle branch area pacing (LBBAP) is an alternative to right ventricular (RV) and biventricular (BiV) pacing in patients scheduled for pace and ablate treatment strategy. However, current delivery sheaths are designed for left-sided implantation, making the right-sided LBBAP lead implantation challenging.

**Case presentation:**

We report a case of a right-sided LBBAP approach via right subclavian vein in a heart failure patient with a persistent left superior vena cava scheduled for pace and ablate treatment of refractory atrial flutter. To enable adequate lead positioning and support for transseptal screwing, the delivery sheath was manually modified with a 90-degree curve at the right subclavian vein and superior vena cava junction to allow right-sided implantation. The distance between the reshaping point and the presumed septal region was estimated by placing the sheath on the body surface under fluoroscopy. With the reshaping of the delivery sheath, we were able to achieve LBBAP with relatively minimal torque. Radiofrequency ablation of the atrioventricular node was performed the next day and the pacing parameters remained stable in short-term follow-up.

**Conclusion:**

With the modification of currently available tools, LBBAP can be performed with the right-sided approach.

## Background

Conduction system pacing (CSP) is an alternative to standard right ventricular (RV) and biventricular (BiV) pacing in patients scheduled for pace and ablate treatment strategy of refractory supraventricular tachycardias. With CSP, we have an option of His bundle pacing (HBP) and left bundle branch area pacing (LBBAP). Small target zone, prolonged procedural times, oversensing of atrial signals, need for RV back-up lead, and unstable capture thresholds, especially after AV node ablation are some limitations associated with HBP [1]. By transeptal lead implantation, LBBAP overcomes some of these limitations making it a more feasible option, especially in the pace and ablate treatment approach [2]. Present limitations of CSP might also reflect the early stage of new technology, since there are very limited tools available for wider clinical adoption of this technique. Current delivery sheaths are designed for left pectoral implantation, making the right-sided LBBAP lead implantation challenging.

We report a case report of a right-sided LBBAP approach in a patient with a persistent left superior vena cava scheduled for pace and ablate treatment of refractory atypical atrial flutter.

## Case presentation

**Fig. 1:**
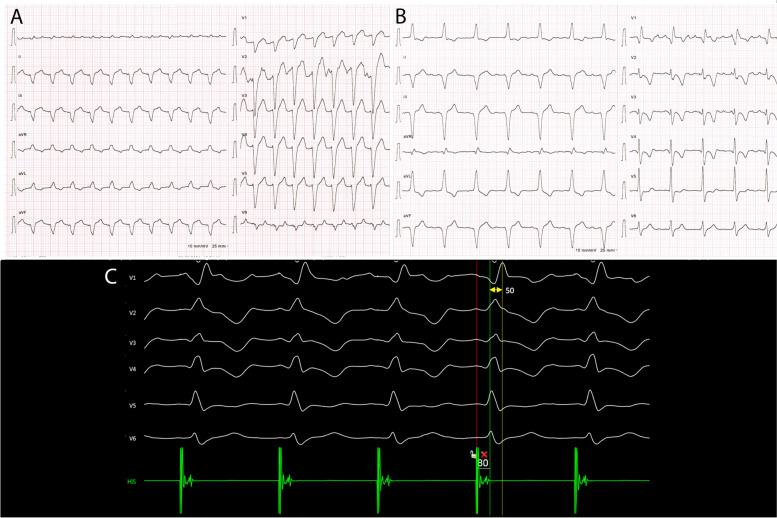
ECG tracings before, during, and after the procedure. **A** A 12-lead ECG tracing at admission showing atypical atrial flutter with left bundle branch block (LBBB) morphology. QRS duration is 150 ms. **B** A 12-lead ECG recorded after the implantation showing paced rhythm (VVI 80 bpm) with correction of LBBB. A distinctive notch in the nadir of the QS complex in V1 can be observed representing left bundle branch area pacing (LBBAP). **C** Intraoperative tracing of precordial leads with intracardiac electrogram in green after trans-septal screwing of the LBBAP lead. With time interval from stimulus to peak of R wave in lead V6 of 80 ms, isoelectric interval on intracardiac electrogram, and the V6-V1 interpeak interval of 50 ms LBB capture was confirmed

Seventy-five-year-old man was admitted to the cardiology department for treatment of drug refractory supraventricular tachycardia and heart failure (HF). He had a history of arterial hypertension and normal coronary angiography. On admission he presented with palpitations, fatigue, legs sweeling, and weight gain of 3 kg in one week. Two weeks prior to admission, the patient underwent cardioversion of atrial flutter, albeit on two antiarrhythmic drugs. Apart from atypical atrial flutter with rapid ventricular rate of 129 bpm, prolonged QRS duration (150 ms) with left bundle branch block (LBBB) morphology was also noted on surface 12-lead ECG (Fig. 1A). Transthoracic echocardiography showed severely enlarged right and left atrium (LAVI 69 ml/m^2^), mildly enlarged left ventricle (EDVI 80 ml/m^2^), reduced left ventricular ejection fraction (LVEF) of 45% with clear signs of LV mechanical dyssynchrony (septal flash and apical rocking). As tachycardia-induced HF along with LBBB-induced cardiomyopathy was suspected, and sinus rhythm restoration after potential catheter ablation was presumed to be low, we decided for pace and ablate strategy. With reduced LVEF and LBBB standard RV pacing was presumed suboptimal. Therefore, LBBAP was opted as a good pacing option while this physiological pacing mode could also deliver correction of LBBB with single lead implantation.

**Fig. 2:**
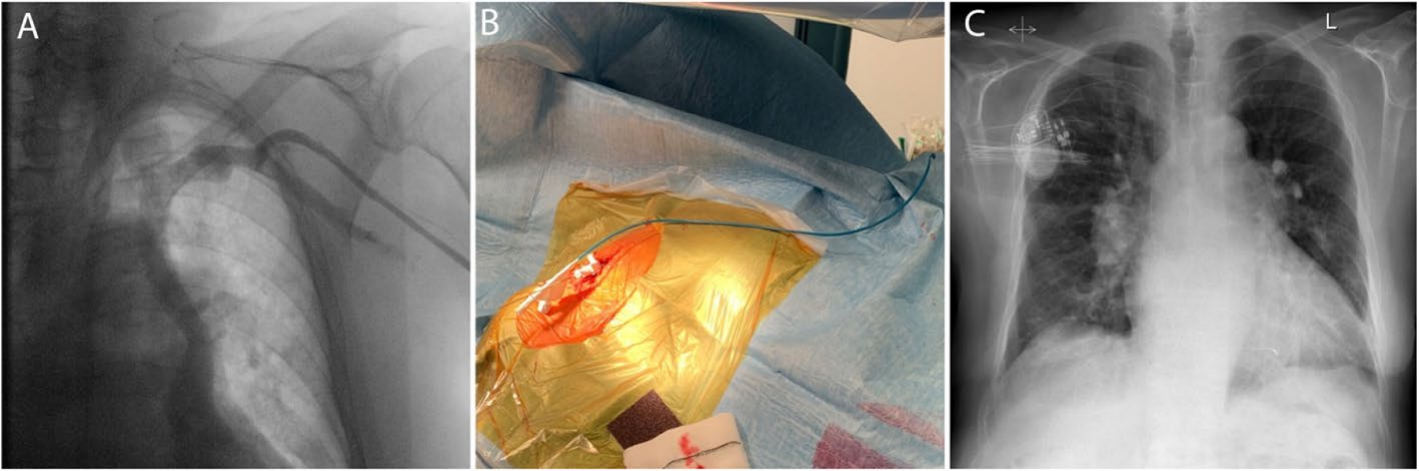
Images of chest x-ray fluoroscopy and manual modification of the C315-HIS delivery sheath. **A** A venography of left subclavian vein showing left superior persistent vena cava. **B** Reshaping point and the presumed septal region was estimated by placing the sheath on the body surface under fluoroscopy. **C** A chest X-ray of the final lead position

**Fig. 3:**
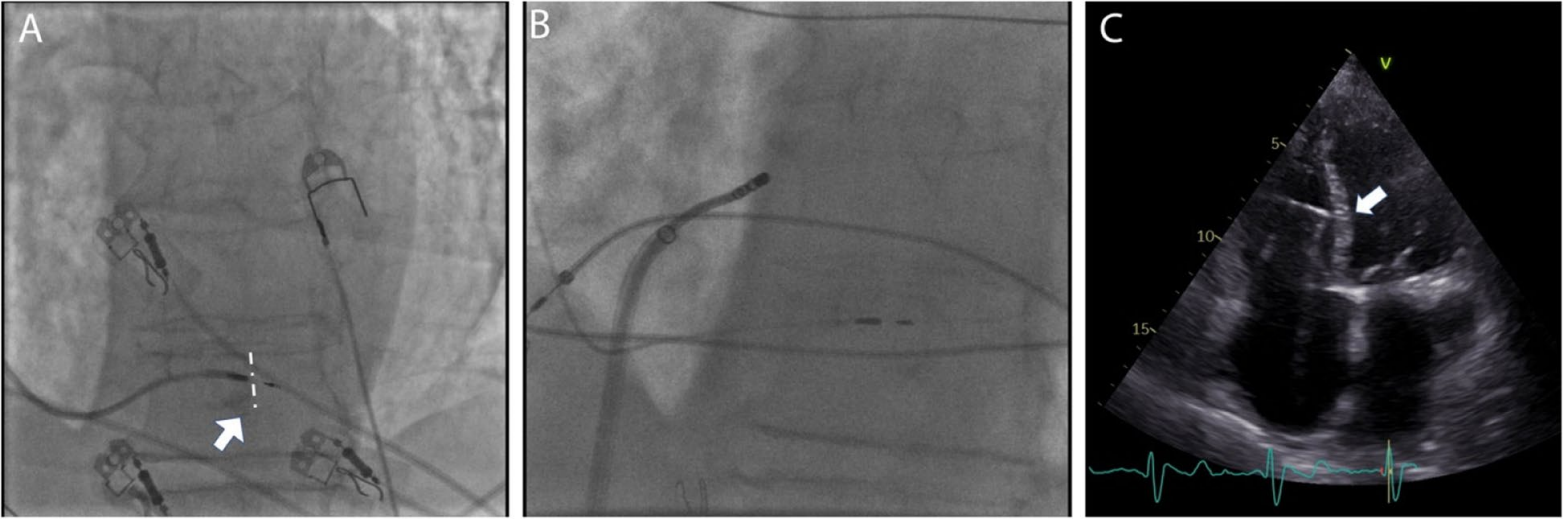
Fluroscopic and echocardiographic images of the left bundle branch pacing (LBBP) lead. **A** LBBP lead in left anterior oblique (LAO) 30-degree fluoroscopic view. A thin layer of contrast can be seen (arrow) outlining the right ventricular septal wall (dashed line), demonstrating the lead depth. **B** Final ablation target site in relation to the pacing lead. **C** Echocardiographic image showing the severely enlarged atria and transseptal position of the pacing lead (arrow)

Venography on the left side showed a persistent left superior vena cava (Fig. 2A). A decision was made to attempt LBBAP lead implantation from the right side. To enable SelectSecure 3830 69 cm pacing lead (Medtronic Inc., Minneapolis, MN, USA) positioning and adequate support for transseptal screwing, the delivery sheath C315-HIS (Medtronic Inc., Minneapolis, MN, USA) was manually modified with dilator in place by creating a 90-degree curve at the right subclavian vein-superior vena cava junction. The distance between the reshaping point and the presumed septal region was estimated by placing the sheath on the body surface under fluoroscopy (Fig. 2B). The sheath was then inserted through the guidewire from the right subclavian vein access to the basal part of the RV septum as previously described. Then, in right anterior oblique 20^0^ the pacing lead was inserted into the delivery sheath to find the initial pacing site, where the V1 lead appeared to be W-shaped. The modification of the C315-HIS catheter allowed perpendicular positioning of the lead tip to the interventricular septum in left anterior oblique 30^0^ with minimal torque. The fluoroscopic image of the final pacing lead implantation site can be viewed in Fig. 3A. Subsequently, the lead was gradually screwed transeptally until unipolar pacing at the site showed a right bundle branch block QRS morphology with a notch in the nadir of the QS complex. With the time interval from stimulus to peak of R wave in lead V6 of 80 ms, isoelectric interval on intracardiac electrogram, and the V6-V1 interpeak interval of 50 ms LBB capture was confirmed (Fig. 1C) [3]. Pacing parameters were excellent: R-wave sensing 15.4 mV, threshold 0.75 V at 0.5 ms, and unipolar pacing impedance 646 Ohm. The lead was connected to dual-chamber pacemaker device in the ventricular port, while a pin was inserted in the atrial port (Fig. 2C). Dual-chamber device was selected in case of any additional lead insertion in the future, e.g. in case of sinus rhythm restoration. The total procedural time was 50 minutes and the fluoroscopy time was 7.4 minutes. During and after the procedure the patient reported no chest pain or other adverse events and there were no ST-T changes present on native 12-lead ECG. AV node ablation with non-irrigated ablation catheter was performed the next day (Fig. 3B). The periprocedural LBBAP lead parameters remained stable. Pacemaker was set to VVIR pacing with 80 bpm rest rate (Fig. 1B).

In a short-term follow-up of 6 months, the LBBAP parameters set to bipolar pacing remained stable (threshold 0.75 V at 0.5 ms, impedance 689 Ohm), the mechanical dyssynchrony was no longer present, and the cardiac function improved (LVEF 60%) (Fig. 3C). The patient also reported improved exercise capacity and no signs or symptoms of HF.

## Discussion and conclusion

With our report of LBBAP in a patient with a persistent left superior vena cava, we showed that this promising CSP technique could be performed even with the right-sided approach by modifying currently available tools. In addition, LBBAP in this patient with refractory supraventricular tachycardia and LBBB seems to be a well-balanced approach while it enables safe AV node ablation and enables resynchronization of the underlying LBBB.

Although reassuring data is emerging of CSP as a viable alternative to conventional RV and BiV pacing in patients with various pacing indications, the availability of additional tools to enable wider clinical adoption is relatively gradual [4–9]. Therefore, different clinical scenarios with challenging anatomical and clinical conditions demand the modification of the presently available tools. Currently available delivery sheaths for CSP are designed for left-sided implantation. Therefore, if a right-sided approach is attempted, the sheath needs constant counter-clockwise rotation. This position has to be maintained carefully during lead positioning. Because of the significant torque required to maintain this position, it is very common for the sheath to rotate back to the right atrial free wall, precluding stability of the catheter and support needed for transseptal LBBAP lead implantation. The described modification of the delivery catheter with the 90-degree curve at the right subclavian vein-superior vena cava junction to enable right-sided implantation of HBP was described by Vijayaraman et al. [10] Using the same approach, we were able to secure adequate perpendicular positioning of the sheath tip to the interventricular septum and sufficient support for transseptal LBBAP lead implantation. Compared to HBP, the LBBAP approach demands greater sheath support as transseptal perforation is needed for successful procedure. As this is a case report, the described modification on the sheath for right-sided approach could not be sufficient in all anatomical variations. Deflectable sheath C304His could also be a feasible option as adjusting the primary curve might be beneficial in some cases. However, compared to the pre-shaped sheath, the adjustable sheath is stiffer and thus it is more difficult to additional modify and create the 90-degree curve at the right subclavian vein-superior vena cava junction. Nonetheless, further development of dedicated tools might improve and facilitate wider clinical adoption of CSP in various pacing indications and challenging anatomical conditions [10].

Apart from atypical anatomical circumstances, our patient presented with possible tachycardia-induced HF due to drug refractory atrial flutter with rapid ventricular rate and underlying LBBB which further deteriorated cardiac function due to mechanical dyssynchrony. The patient also had severely enlarged left atrium (LAVI 69 ml/m^2^). A meta-analysis of 21 observational studies showed that patients with arrhythmia recurrence had a higher mean LAVi [11]. Furthermore, Shin et al. showed that LAVi of 34 ml/m^2^had a 70% sensitivity and specificity of 91% for arrhythmia recurrence [12]. Therefore, the probability of successful catheter ablation was deemed low and pace and ablate strategy was opted. In the sense of pace and ablate strategy and underlying LBBB, LBBAP seems to be the optimal CSP approach in our patient [13–16]. LBBAP could potentially overcome some limitations of HBP, such as higher capture thresholds, distal location of the conduction block with wide QRS, additional RV backup lead, and technical challenges with shorter procedural time [1]. In addition, AV node ablation is safer to perform when the pacing lead is inserted further away from the ablation site. On the other hand, compared to standard BiV pacing, where anatomy of the coronary sinus tributeries greatly impacts the degree of resynchronization and procedural times, LBBAP could provide even better resynchronization in proximal conduction abnormalities with LBBB, as in our case [17, 18].

With the modification of currently available tools, LBBAP technique can be performed with the right-sided approach. Further improvement of dedicated tools might improve and facilitate wider clinical adoption of CSP in various pacing indications and challenging anatomical conditions. Further studies are needed to evaluate the feasibility, efficacy, and safety of right-sided approach for LBBAP implantation.

## Data Availability

We will share the data on reasonable request to the corresponding author.

## Abbreviations

AV: Atrioventricular
BiV: Biventricular
CSP: Conduction system pacing
EDVI: End diastolic volume index
HBP: His bundle pacing
HF: Heart failure
LAVI: Left atrial volume index
LBBAP: Left bundle branch area pacing
LBBB: Left bundle branch block
LVEF: Left ventricular ejection fraction
RV: Right ventricle
